# Hearing screening outcomes and otoscopic findings among primary school children in Southwest Ethiopia: A cross-sectional study

**DOI:** 10.4102/sajcd.v73i1.1175

**Published:** 2026-07-22

**Authors:** Gugsa N. Germossa, Linn S. Mork, Ebissa B. Kebede, Konstantinos Antypas, Kora Tushune, Tron V. Tronstad, Arne H. Eide, Vinay S. Nagaraj, Tone Øderud

**Affiliations:** 1 School of Nursing, Faculty of Health Sciences, Institute of Health, Jimma University, Jimma, Ethiopia; 2 Department of Neuro Medicine and Movement Sciences, Audiology Group, Norwegian University of Science and Technology, Trondheim, Norway; 3 Department of Health Research, SINTEF Digital, Oslo, Norway; 4 Departments of Health Policy and Management, Faculty of Public Health, Institute of Health, Jimma University, Jimma, Ethiopia; 5 Ministry of Education, Addis Ababa, Ethiopia; 6 Department of Neuro Medicine and Movement Sciences, Norwegian University of Science and Technology, Trondheim, Norway

**Keywords:** hearing screening, otoscopic findings, schoolchildren, primary schools, Jimma, Ethiopia

## Abstract

**Background:**

Hearing loss among schoolchildren is a major public health concern that negatively affects language, speech, learning and social development. However, evidence on hearing screening outcomes among schoolchildren in low- and middle-income countries (LMICs) remains limited.

**Objectives:**

This study aimed to determine hearing screening outcomes and otoscopic findings among primary schoolchildren in Southwest Ethiopia.

**Method:**

A school-based cross-sectional study was conducted among 396 primary schoolchildren in Jimma Town. A pragmatic school-based sampling approach was used to select participants. A two-stage screening process was implemented, consisting of pure-tone audiometry (at 500 Hz, 1000 Hz, 2000 Hz and 4000 Hz) followed by otoscopic examination for children who failed the hearing screening. Descriptive statistics, proportions and 95% confidence intervals were calculated using MATLAB version 2024b.

**Results:**

The proportion of children who failed the hearing screening (hearing level ≥ 25 dB at one frequency in at least one ear) ranged from 13% to 22% across four schools and reached 68.9% in the fifth school. Impacted earwax was the most common otoscopic finding, accounting for nearly 34%.

**Conclusion:**

A substantial proportion of children failed the hearing screening, mainly due to preventable and treatable conditions. The findings suggest the need for school-based hearing screening programmes, ear hygiene education and improved access to ear care services in resource-limited settings.

**Contribution:**

This study provides valuable data on hearing screening outcomes and otoscopic findings in schoolchildren in southwest Ethiopia and highlights the importance of regular hearing screenings, education on ear hygiene and improved access to hearing care.

## Introduction

Hearing loss (HL) in children is a serious but often overlooked public health issue in low-resource areas, causing major delays in development and education. Globally, an estimated 34 million children live with hearing loss (World Health Organization [WHO], 2025), with projections indicating that more than 2.5 billion people could be affected by 2050 (WHO, 2021). In low- and middle-income countries (LMICs), particularly in sub-Saharan Africa, most children with hearing loss are neither diagnosed nor treated and do not get the help they need (Abdalla & Omar, 2011; Mahomed-Asmail et al., 2016; Waterworth et al., 2022). This is largely because of a severe shortage of ear, nose and throat (ENT) specialists, audiologists and hearing services, as well as low awareness and health literacy about ear and hearing care (Mahomed-Asmail et al., 2016; Mulwafu et al., 2017; WHO, 2021). Hearing loss in children impacts language and cognitive development, academic performance, social inclusion, quality of life and future employability, and they often have lower reading scores, need special education and are more likely to repeat classes (Le Clercq et al., 2020; Lieu, 2020; WHO, 2025). Early identification of hearing loss is key to implementing effective hearing care and requires accessible hearing screening for the detection of those who are at risk (WHO, 2025). School-based hearing screening programmes have been recommended as a practical and cost-effective first step in resource-constrained environments (Bright, 2011; Dawood et al., 2021). Such programmes utilising digital hearing screening can detect both temporary and permanent hearing losses and facilitate timely management of preventable causes such as impacted earwax and middle-ear infections. Studies from South Africa have demonstrated the feasibility and value of school-based hearing screening programmes (Govender et al., 2015; Mahomed-Asmail et al., 2016; Phanguphangu, 2017; Pierce & Stemela-Zali, 2025). A person is said to have hearing loss if they cannot hear as well as someone with normal hearing (WHO, 2025). WHO defines hearing with reference to hearing threshold levels of 20 dB or better in both ears (WHO, 2025). Furthermore, disabling hearing loss refers to hearing loss greater than 30 dB in the better hearing ear in children with an average of 500 Hz, 1000 Hz, 2000 Hz and 4000 Hz (Olusanya et al., 2019). Hearing loss may be permanent or temporary, and it can range from mild to profound, and it can affect one or both ears. Hearing loss can be categorised into three main types: conductive hearing loss (CHL), sensorineural hearing loss (SNHL) and mixed hearing loss, which is a combination of CHL and SNHL, each with unique characteristics and causes, especially among primary schoolchildren (Madell & Flexer, 2014). Conductive hearing loss occurs when sound waves cannot efficiently travel through the outer and middle ear to the inner ear. Ear infections, impacted earwax and a foreign body blocking the ear canal and abnormalities in the outer and/or middle ear may cause CHL in children (Robson, 2023). Sensorineural hearing loss arises from damage or dysfunction of the inner ear or the auditory nerve. In children, the most common causes of SNHL include genetic factors, congenital infections, exposure to ototoxic medications and noise-induced hearing loss (Korver et al., 2017). While CHL might be preventable, SNHL is often permanent, requiring appropriate interventions that may include hearing aids, cochlear implants or other assistive listening devices (Kleinjung & Londero, 2024). Evidence on the prevalence of hearing loss among primary schoolchildren in sub-Saharan Africa remains limited. Reported prevalence of hearing loss varies widely depending on screening protocols and thresholds used (Moepeng et al., 2021; Shinn et al., 2021). Data on school-based hearing screening performance covering the whole of Ethiopia are particularly scarce (Birhanu et al., 2021; Haile et al., 2023; Meshesha et al., 2025). Therefore, this study aimed to conduct school-based hearing screening in primary schools to determine hearing screening outcomes among primary schoolchildren in Jimma Town, Southwest Ethiopia. The specific objectives were: (1) to measure the rate of children who failed pure-tone audiometry at different thresholds and (2) to identify common otoscopic findings among children who failed screening.

## Research methods and design

### Study design and study setting

This school-based cross-sectional study was conducted at five primary schools in Jimma Town to determine hearing screening outcomes and otoscopic findings. Jimma is one of the historic and culturally diverse towns in Oromia. It is widely recognised for being the origin of coffee Arabica and is surrounded by fertile highlands that support rich agricultural activity and biodiversity. The town serves as an important commercial and educational centre in the region, with a population drawn from diverse ethnic and cultural backgrounds. The town is located at, approximately 352 km from the capital city, Addis Ababa. Primary schools in Ethiopia cover Grades 1–6 and can be either public, private or run by non-governmental organisations (NGOs). All these school types were represented in our study.

### Population and sampling

A pragmatic school-based sampling strategy was employed to capture diversity across different types of primary schools represented in Jimma Town. Four schools were selected systematically from available primary schools to ensure broad representation, including two public schools, one private school and one NGO-run school. The fifth school, a public school serving a highly vulnerable population, was added at the school principal’s request. Although its inclusion was non-systematic, it was retained to ensure representation of an otherwise under-represented subgroup, and such pragmatic decisions are common in field-based research (Chan et al., 2021). All second-grade students within the five schools were invited to participate to maximise feasibility and participation. Of the 1330 eligible students, 396 provided assent and parental consent. Formal *a priori* power calculations were not conducted. The non-systematic inclusion of the fifth school is acknowledged as a potential source of selection bias. As long as the children provided assent and parental consent, the exclusion criterion is not specified in this case, as the study aimed to include all eligible participants in grade two. Children within the selected schools were approached for participation upon obtaining permission from school authorities, parents and guardians.

### Instruments and data collection procedures

Before starting the screening, participants were informed and interviewed face-to-face by a member of the research team. Participants’ characteristics were registered. Hearing screening was conducted in an ambient room (vacant classroom or offices) at each primary school. The study used a two-stage hearing screening process. Initially, pure-tone audiometry was conducted, followed by otoscopic examination for those who failed the audiometry. Audiometry was performed using a battery-driven audiometer (Micromate 304, Otometrics) at frequencies of 500 Hz, 1000 Hz, 2000 Hz and 4000 Hz using Sennheiser HDA 200 headphone, following the modified Hughson-Westlake procedure. The audiometer was calibrated according to the International Organization for Standardization (ISO) 389-1 standards (Carhart & Jerger, 1959; ISO, 2010; Sennheiser, 2025). The headphones were sanitised between participants. Oral instructions and demonstrations were provided to the children in their local language (Oromo or Amharic). All diagnostic equipment was portable, chargeable and battery-operated. Ambient noise levels were monitored continuously using a calibrated sound level metre and maintained at ≤ 40 dB (A) during testing. School-based hearing screening is typically conducted in enclosed, unoccupied and furnished classrooms, where ambient noise levels commonly range from 30 dB(A) to 64 dB(A), often exceeding the recommended maximum of 35 dB(A) for audiometric testing (McPherson et al., 2010). Although lower noise levels (≤ 30 dB[A]) are ideal, a ≤ 40 dB(A) was accepted as a practical threshold commonly used in field-based screening in resource-limited settings (McPherson et al., 2010). To minimise interference, headphones with sound attenuating properties were used. During the hearing screening, participants were asked to raise their hands to the side where they heard the sound. Normal hearing was defined as hearing thresholds up to 25 dB HL, and participants with a threshold equal to or greater than 25 dB HL for one frequency, 0.5 kHz, 1 kHz, 2 kHz and 4 kHz in at least one ear, were categorised as having failed the hearing screening (Olusanya et al., 2019; WHO, 2021). Children who failed the hearing screening underwent otoscopic examination by ENT physicians for potential ear infection, earwax and foreign bodies. All interventions, including infection treatment, foreign body removal and ear wax removal, were performed after pure-tone audiometry screening and otoscopic examination. Medication was provided according to the ear infection treatment protocol in Ethiopia. Foreign bodies were removed for those identified as having foreign bodies. Earwax was removed using ear irrigation with lukewarm water (Propulse Ear Irrigator – Purple Lid). These interventions were conducted on site at the local schools by ENT nurses and physicians. Children identified with severe infection, earwax severely blocking the ear canal, retained foreign bodies and suspected SNHL were referred to the ENT clinic for further evaluation. The project covered the expenses of the assessment, diagnosis and treatment.

### Data analysis

After collecting the data, it was entered into Microsoft Excel for initial organisation and preparation. The dataset was then imported into MATLAB 2024b (24.2.0.2833386), for further analysis. Descriptive statistics, including frequencies, percentages and 95% confidence interval (CI), were used to provide an overview of the sample’s characteristics and distributions of the variables. As the study was descriptive in nature, no inferential statistical tests were conducted.

### Ethical considerations

This study was conducted in accordance with the Declaration of Helsinki and ethical principles for research involving children and focused on ensuring the well-being, privacy and dignity of all participants involved. Ethical approvals were obtained from the relevant authorities in Norway and Ethiopia. In Norway, the approvals were granted by the Norwegian National Committees for Research Ethics (REK) under reference number 522539. The study was also approved by the Jimma University Institutional Review Board (reference JUIRB 23/22). Permissions were obtained from the Jimma Town Education Office and school principals. Parents or guardians were informed about the study orally by the school principals and by written letter in their local language (Oromo and Amharic). Parents or guardians of participating children provided written informed consent 1 week prior to the study. Screening was performed only for those children who volunteered to participate. Children with earwax obstructing the ear canal received appropriate earwax removal. Those diagnosed with mild external or middle-ear infections were treated with antibiotics. Children with extensive ear infections were referred to Jimma University Medical Center for further medical management. In addition, children suspected of having SNHL were referred to Oda Hulle Hospital (a private hospital with better facilities for assessing SNHL in Jimma Town) for comprehensive diagnostic evaluation. The project covered all costs related to assessment, diagnosis, referral and treatment services provided during the study.

## Results

### Sample characteristics

The study included 396 children from five schools in Jimma Town. Participants consisted of 174 (43.9%) males. The mean age was 9.11 years (±1.71 standard deviation [s.d.]), with almost half, 195 (49.2%), falling within the age range of 7–8 years. School participation varied, with School ‘A’ contributing 26.8% of participants (Table 1).

**TABLE 1:** Participants’ characteristics among primary schoolchildren in Jimma (*N* = 396).

Variables	Frequency (*n*)	%
**Sex**		
Male	174	43.91
Female	222	56.10
**Age (years)**		
7–8	195	49.20
9–11	134	33.80
> 11	67	16.90
**School**		
A	106	26.80
B	48	12.10
C	54	13.60
D	83	21.00
E	105	26.50

### Screening outcomes

Overall, 31.6% (95% CI: 27.0% – 36.1%) of children failed the hearing screening at the ≥ 25 dB threshold in at least one ear. Failure rates varied markedly across the schools, ranging from 13.3% to 22.2% across four schools and 68.9% in School ‘A’. Low frequency at 500 Hz showed failure in 28.8% (95% CI: 24.2% – 33.4%) of children, while high frequency at 2 kHz and 4 kHz showed failure in 24% (95% CI: 20.2% – 28.0%). About 7.6% (95% CI: 5% – 10.2%) showed low-frequency failure exclusively, compared to 2.8% (95% CI: 1.5% – 5.0%) with high-frequency failure only. These findings represent screening outcomes rather than confirmed diagnostic hearing loss (Table 2).

**TABLE 2:** Hearing screening failure rates in schoolchildren at various thresholds.

Variables	Hearing level ≥ 25		Hearing level ≥ 30		Hearing level ≥ 35		Hearing level ≥ 40		Hearing level ≥ 45	
	%	95% CI	%	95% CI	%	95% CI	%	95% CI	%	95% CI
Overall screening failure	31.6	27, 36.1	20.2	16.2, 24.2	11.4	8.2, 14.5	7.6	5, 10.2	4.8	2.7, 6.9
Low frequency (at 500 Hz)	28.8	-	18.7	-	10.4	-	7.1	-	3.8	-
High frequency (at either 2 kHz or 4 kHz)	24.0	-	13.6	-	7.8	-	4.5	-	3.5	-
Only low frequency	7.6	-	6.6	-	3.5	-	3.0	-	1.3	-
Only high frequency	2.8	-	1.5	-	1.0	-	0.5	-	1.0	-

CI, confidence interval.

Note: CI is the 95% confidence interval. Low-frequency failure was defined as a threshold ≥ 25 dB at 500 Hz, and high-frequency failure as a threshold ≥ 25 dB (at either 2 kHz or 4 kHz). Differences are not statistically evaluated; interpret variability with caution.

A thorough analysis of hearing screening thresholds across various schools and age groups revealed variability. For instance, School A had the highest failure rate (68.9%). In age-group analysis, 84.6% children aged 7–8 years have hearing thresholds of ≤ 30 dB HL. In comparison, 35% of the 9–10-year age group failed the screening at 25 dB HL (Table 3 and Table 4).

**TABLE 3:** Hearing screening outcomes among primary schoolchildren in Jimma using frequency limits of 25 dB.

Variables	Hearing screening threshold					
	< 25 dB			≥ 25 dB		
	Frequency (*n*)	%	95% CI	Frequency (*n*)	%	95% CI
**School**						
A	33	31.1	22.5, 40.9	73	68.9	59.1, 77.5
B	41	85.4	72.2, 93.4	7	14.6	6.1, 27.8
C	42	77.8	64.4, 88.0	12	22.2	12.0, 35.6
D	72	86.7	77.5, 93.2	11	13.3	6.8, 22.5
E	83	79.0	70.0, 86.3	22	21.0	13.6, 30.0
**Age (years)**						
7–8	144	73.8	67.1, 79.9	51	26.2	20.1–33.0
9–11	86	64.2	55.4, 72.3	48	35.8	27.7, 44.6
> 11	41	61.2	48.5, 72.9	26	38.8	27.1, 51.5
**Sex**						
Male	139	79.9	73.2, 85.6	35	20.1	14.4, 26.9
Female	132	59.5	52.8, 66.0	90	40.5	34.0, 47.3

CI, confidence interval.

**TABLE 4:** Hearing screening outcomes among primary schoolchildren in Jimma using frequency limits of 30 dB hearing loss.

Variables	Hearing screening threshold					
	≤ 30 dB HL			> 30 dB HL		
	Frequency (*n*)	%	95% CI	Frequency (*n*)	%	95% CI
**School**						
A	54	50.9	41.1, 60.8	52	49.1	39.2, 59.0
B	47	97.9	88.9, 100.0	1	2.1	0.1, 11.1
C	48	88.9	77.4, 95.8	6	11.1	4.2, 22.6
D	75	90.4	81.9, 95.8	8	9.6	4.3, 18.1
E	92	87.6	79.8, 93.4	13	12.4	6.8, 20.2
**Age (years)**						
7–8	165	84.6	78.8, 89.4	30	15.4	10.6, 21.2
9–11	100	74.6	66.4, 81.7	34	25.4	18.3, 33.6
> 11	51	76.1	64.1, 85.7	16	23.9	14.3, 35.9
**Sex**						
Male	153	87.9	82.1, 92.4	21	12.1	7.6, 17.9
Female	163	73.4	67.1, 79.1	59	26.6	20.9, 32.9

Note: Differences across schools, age groups and sex were not statistically tested and should therefore be interpreted cautiously.

HL, hearing loss; CI, confidence interval.

### Otoscopic finding

Among children who failed the hearing screening, impacted earwax was the most common otoscopic finding, accounting for almost 34% of the cases. Foreign bodies were identified in four children, while middle-ear infections were observed in three children. Other otoscopic abnormalities, including tympanic membrane perforation, were observed in two of the cases. About 59% of children who failed the screening had normal otoscopic findings (Table 5). This may indicate the presence of SNHL, transient hearing threshold shifts, middle-ear dysfunction not detectable through otoscopy alone or false-positive screening results.

**TABLE 5:** Distribution of otoscopic findings by school (*N* = 124).

Otoscopic finding	School											
	A		B		C		D		E		Total	
	*n*	%	*n*	%	*n*	%	*n*	%	*n*	%	*n*	%
Normal	44	61.1	6	85.7	3	25.0	5	45.5	15	68.2	73	58.9
Impacted earwax	22	30.6	-	-	7	58.3	6	54.5	7	31.8	42	33.9
Foreign body	1	1.4	1	14.3	2	16.7	-	-	-	-	4	3.2
Middle-ear infection	3	4.2	-	-	-	-	-	-	-	-	3	2.4
Other (ruptured tympanic membrane)	2	2.8	-	-	-	-	-	-	-	-	2	1.6

## Discussion

This study reports the outcome of hearing screening rather than confirmed hearing loss, as diagnostic audiometry in a clinical setting, tympanometry and bone conduction testing were not performed. All findings should be interpreted as screening results. The study showed a proportion of children who failed hearing screening among primary school students in Jimma Town. The screening failure rate varies by age, gender and school setting. A substantial proportion of children (13.3% – 22.2%) in four schools and 68.9% in School ‘A’ failed the hearing screening at the 25 dB HL threshold. This raises significant public health concerns and underscores the necessity for enhanced awareness and intervention strategies in the population, as it could significantly impact the schoolchildren’s social interaction, communication and overall quality of life (WHO, 2021). The distinction between low-frequency (28.8%) and high-frequency (24%) screening failures indicates that low-frequency failures are more common, with implications for auditory processing and communication skills, especially in educational settings (Škerková et al., 2023). Although 7.6% of participants exhibited low-frequency failure exclusively, compared with 2.8% showing only high-frequency failure, this pattern may partly reflect exposure to persistent low-frequency background noise in the study environment. Nevertheless, because noise-induced hearing impairment typically affects higher frequencies, other factors – such as middle-ear conditions or measurement artefacts – could also contribute to this finding. This finding aligns with the existing literature emphasising the impact of low-frequency sounds in everyday communication, particularly in noisy environments (Buono et al., 2021). Targeted screening and early intervention programmes could help address these issues. The hearing screening failure rate observed in our study is higher than the global estimates, ranging from 1% to 5% for children with mild to moderate hearing loss, from 7% to 17% in Tanzania (Ertzgaard et al., 2020), 11.9% in Ghana (Osei et al., 2018), 9.2% in Egypt (Morgan et al., 2021), and the global average, which is 20% (WHO, 2023). The observed difference between the global figures and the findings from Jimma may reflect contextual or environmental factors that warrant further investigation. However, the current study’s findings are lower than those conducted in another part of Ethiopia, where the prevalence was 34% in Addis Ababa (Haile et al., 2023). This variation could be attributed to differences in design and sample size. Several factors may be associated with higher screening failure rates, including limited access to healthcare, environmental factors and socio-economic challenges (Adadey et al., 2022; Lee et al., 2024; Mo et al., 2024; Mulwafu et al., 2017). Limited access to healthcare services, particularly to ENT specialists and audiologists, can result in untreated or poorly managed ear conditions. Environmental factors, such as increased exposure to noise in overcrowded school environments, could also contribute to screening failure. Moreover, socio-economic barriers, including financial constraints, may prevent children from receiving preventive care or timely treatment for ear conditions, increasing the risk of screening failure. The findings suggest unmet hearing health needs and the need for targeted interventions to address hearing health in primary schoolchildren; however, a confirmatory diagnostic assessment is required to determine the true prevalence of HL. This includes improving access to healthcare, raising awareness and implementing preventive measures to reduce the burden on families and the healthcare systems (Khoza-Shangase, 2021). The high screening failure may also reflect a lack of early childhood hearing screening programmes in the current study. In many high-income countries, universal newborn hearing screening programmes have been implemented, leading to earlier identification and intervention for hearing loss. However, in low-income settings like Ethiopia, such programmes are often lacking, which may partly account for the higher prevalence observed in the current study. The other finding is differences in hearing screening participation rates across the five schools in the study. The highest screening failure was reported in School ‘A’ (68.9%), while Schools ‘B’ and ‘D’ had much lower prevalence, at 14.6% and 13.3%, respectively. These differences may reflect exposure to higher noise levels, limited access to healthcare and the family’s socio-economic status. Exposure to higher levels of noise may also contribute to noise-induced hearing loss. Noise-induced hearing loss is a well-documented phenomenon that can occur even at relatively low noise levels if the exposure is prolonged (Basner et al., 2014). School ‘A’ has a relatively overcrowded class environment, which might result in elevated noise levels and, combined with potentially limited access to hearing care services, could explain the significantly higher rates of screening failure. However, this study did not directly measure noise exposure or confirm noise-induced hearing loss. Differences in healthcare access across schools, including ear and hearing care, could be another factor explaining variation between schools. Schools with a higher screening failure rate may serve communities that face more significant barriers to accessing healthcare, including financial constraints, geographic isolation or a lack of awareness about hearing health. The revealed age-related pattern is consistent with previous findings showing that older children exhibit higher rates of screening failures. This age-related pattern is consistent with the idea that hearing loss can be cumulative, with the risk increasing as children age due to prolonged exposure to environmental risks and untreated ear conditions (Charles Stewart Mott Children’s Hospital [CSMCs], 2024). The observed increase in the screening failure rates with age may be due to the gradual development of chronic conditions, such as recurrent ear infections or noise-induced hearing loss (Teele et al., 1989). Additionally, older children may be more exposed to environmental noise, such as through increased participation in extracurricular activities, attendance at noisy events or use of personal audio devices (e.g. headphones) at higher volumes, all of which can contribute to SNHL over time (Shargorodsky et al., 2010). It may also reflect cumulative exposure to risk factors over time, although causality cannot be inferred from the current cross-sectional design. The study also identified higher screening failure rates among females (40.5%) compared to males. This finding is somewhat paradoxical, as other studies from Lusaka, Zambia (Hapunda et al., 2020) and Cape Town, South Africa (Kuschke et al., 2020) have reported that males are generally at higher risk of screening failure than females in the general population, and requires further investigation. However, the higher rates of screening failure among females in the study may reflect contextual or unmeasured factors that were not directly assessed in this study. Potential biological differences, such as hormonal variations, could also affect susceptibility to hearing loss, though this area requires further research (McFadden, 2014). Possible sociocultural or environmental explanations warrant exploration in future studies. Regarding the otoscopic findings, impacted earwax was the most common reversible condition identified, but if left untreated, it can lead to conductive hearing loss by blocking sound from reaching the eardrum. This highlights the need for improved ear hygiene and access to basic healthcare services in the community. Public health interventions addressing ear hygiene practices, particularly in schools, could significantly reduce the burden of hearing loss caused by impacted earwax (Schwartz et al., 2017). The WHO estimated that 60% of hearing loss is preventable (Mo et al., 2024). This finding supports the importance of preventive ear and hearing care services in school settings. According to the WHO, improving living conditions, promoting good personal hygiene and screening for early detection of hearing loss can all help reduce the likelihood of hearing loss (WHO, 2016). While infections accounted for a relatively small proportion of hearing loss, it is important to note that untreated ear infections can lead to more severe complications, including permanent hearing loss. This finding underscores the need for early diagnosis and treatment of ear infections to prevent long-term hearing damage in children.

### Limitations

Several limitations should be considered when interpreting these findings because this was a hearing screening and not a diagnostic assessment. Despite noise control efforts, field conditions may have influenced low-threshold (25 dB HL) results. The high rate of normal otoscopy (59%) suggests either SNHL or possible false positives due to environmental and/or technical factors. The study was conducted in only five primary schools, which limits the generalizability of the findings. We did, however, ensure that all school types were represented in the sample and assume that the results do provide a valid picture of the situation among primary school students in Jimma Town. Pure-tone audiometry served as a preliminary hearing screening tool, primarily identifying children with potential hearing loss rather than providing a comprehensive audiological evaluation. Relying solely on otoscopic examination may have overlooked other underlying causes of hearing loss, such as sensorineural or central auditory conditions, which this method cannot detect. The study did not account for genetic factors, which can play a significant role in hearing loss and may have contributed to the screening failure rate observed. The study did not fully explore the potential influence of cultural and socio-economic factors on hearing loss, thereby limiting its ability to provide a deeper understanding of disparities in prevalence across populations. Furthermore, the study’s inclusion of a fifth participating school based on a self-reported request raises potential selection bias, as the reasons for voluntary participation may not be representative of schools selected through a controlled process, potentially affecting the overall representativeness of the findings. These limitations suggest that further research is needed to provide a more holistic knowledge of paediatric hearing loss in this context. No inferential statistical analyses were conducted; therefore, observed differences between schools, age groups and sex should not be interpreted as statistically significant associations.

### Implications

The results obtained from this study emphasise the importance of developing a comprehensive public health strategy to enhance ear and hearing conditions among primary school pupils in Jimma and other similar regions. Due to the fact that the majority of failures to pass the screening tests were related to issues that can be prevented and cured through proper care and hygiene, including impacted earwax, a hearing screening programme, along with ear hygiene education in primary schools, is likely to help decrease the number of cases of hearing difficulties. This approach must involve training teachers on ear health and hygiene, introducing relevant educational programmes in the school curriculum and developing procedures for referral to specialised services for treating ear problems. In highly affected schools, adjustments must also be made concerning the environment, for example, noise reduction measures taken. Collaboration among the education ministry, the Ministry of Health, NGOs and local health institutions must be achieved at the policy level to implement successful and sustainable hearing screening initiatives. Future research should pay attention to the cost-effectiveness analysis of hearing screening in Ethiopia and full diagnostics and audiological examinations.

### Recommendations

School-based hearing screening programmes in similar settings should be strengthened through collaboration between schools, health institutions and government agencies. Integration of ear hygiene education, teacher training and systematic referral pathways to ENT and/or audiology services could improve early identification and management. Future research should be conducted using full diagnostic audiology and randomised or cluster randomised sampling to establish more accurate prevalence estimates of permanent hearing loss. The cost-effectiveness of screening programmes in Ethiopian primary schools would also be valuable for policy decisions.

## Conclusion

This hearing screening study found that a substantial proportion of primary schoolchildren in Jimma Town demonstrated elevated hearing thresholds (≥ 25 dB) during assessment, ranging from 13% to 22% across four schools and 68.9% in the fifth school. Early detection is crucial for addressing hearing difficulties, as untreated hearing loss can profoundly affect language acquisition, educational outcomes and social development. The findings from this study underscore the urgent need for a multi-tiered approach to improve ear and hearing health among schoolchildren in Jimma and similar low-resource settings. Children who fail screening require confirmatory diagnostic evaluations, including speech audiometry, bone conduction testing and tympanometry, as preliminary results cannot distinguish between temporary impairments and permanent hearing difficulties. Given that reversible conditions such as impacted earwax accounted for a substantial proportion of screening failures, preventive and early intervention measures are highly warranted. This measure includes integrating ear hygiene education into school curricula, raising teachers’ awareness of safe wax removal techniques and establishing annual screening with clear referral protocols. Environmental factors also warrant attention by introducing noise reduction strategies, such as sound-absorbing classroom materials, which could mitigate both false positives and genuine noise-induced damage. Critically, these efforts must align with resource realities in low-income settings. Partnerships with NGOs and government agencies should focus on subsidising diagnostic services and deploying mobile clinics to expand rural access. However, resource allocation decisions should not rely solely on screening outcomes without confirmatory diagnostic evaluation, which may be inflated by environmental artefacts or reversible conditions; hence, pilot programmes validating cost-effective diagnostic pathways are essential to ensure equitable and efficient use of limited resources.
